# High magnetic field induced changes of gene expression in arabidopsis

**DOI:** 10.1186/1477-044X-4-7

**Published:** 2006-12-22

**Authors:** Anna-Lisa Paul, Robert J Ferl, Mark W Meisel

**Affiliations:** 1 Department of Horticultural Sciences and The Biotechnology Program, University of Florida, Gainesville, FL 32611-0690, USA; 2 Department of Physics and National High Magnetic Field Laboratory, University of Florida, Gainesville, FL 32611-8440, USA

## Abstract

**Background:**

High magnetic fields are becoming increasingly prevalent components of non-invasive, biomedical imaging tools (such as MRI), thus, an understanding of the molecular impacts associated with these field strengths in biological systems is of central importance. The biological impact of magnetic field strengths up to 30 Tesla were investigated in this study through the use of transgenic Arabidopsis plants engineered with a stress response gene consisting of the *alcohol dehydrogenase *(*Adh*) gene promoter driving the β-glucuronidase (GUS) gene reporter.

**Methods:**

Magnetic field induced *Adh*/GUS activity was evaluated with histochemical staining to assess tissue specific expression and distribution, and with quantitative, spectrofluometric assays to measure degree of activation. The evaluation of global changes in the Arabidopsis genome in response to exposure to high magnetic fields was facilitated with Affymetrix Gene Chip microarrays. Quantitative analyses of gene expression were performed with quantitative real-time polymerase-chain-reaction (qRT-PCR).

**Results:**

Field strengths in excess of about 15 Tesla induce expression of the *Adh*/GUS transgene in the roots and leaves. From the microarray analyses that surveyed 8000 genes, 114 genes were differentially expressed to a degree greater than 2.5 fold over the control. These results were quantitatively corroborated by qRT-PCR examination of 4 of the 114 genes.

**Conclusion:**

The data suggest that magnetic fields in excess of 15 Tesla have far-reaching effect on the genome. The wide-spread induction of stress-related genes and transcription factors, and a depression of genes associated with cell wall metabolism, are prominent examples. The roles of magnetic field orientation of macromolecules and magnetophoretic effects are discussed as possible factors that contribute to the mounting of this response.

## Background

The possibility that strong, static (non-gradient) magnetic fields might have an influence on biological processes has been discussed for many years [1-6], including reports that implicate high magnetic fields in alterations of the cleavage plane during cell division [7-9] and other cellular disorders [10]. Nevertheless, the common viewpoint is that presently achievable static magnetic fields do not have a lasting effect on biological systems [3-5]. Indeed, magnetic resonance imaging (MRI), utilizing static magnetic fields up to 12 Tesla, is a powerful tool for non-invasive *in vivo *imaging at the molecular level [11,12]. The demands for more precise *in vivo *imaging have driven the field strengths progressively higher, approaching 20 Tesla [13], yet information regarding the biological impact of exposing metabolically active cells to fields of this magnitude is limited. Herein, we report the effect of high magnetic fields on the gene expression profile of the plant Arabidopsis (*Arabidopsis thaliana*).

This research was initially driven by an interest in using magnetic levitation as a ground-based model for the effects of a reduced gravity environment on plant gene expression. The utility of using magnetic levitation to mimic a reduced gravity environment has been explored in a variety of systems [14-18]. Our research efforts to take advantage of this venue were initiated with the use of transgenic Arabidopsis that had been engineered with a gene reporter shown to be induced by the spaceflight environment and named TAGES – Transgenic Arabidopsis Gene Expression System [19]. The TAGES Arabidopsis plants are engineered with the GUS (β-glucuronidase) reporter gene driven by the *alcohol dehydrogenase *(*Adh*) gene promoter, which responds to a variety of environmental stresses [20] that initiate transcription of the GUS reporter gene. The GUS expression can be monitored qualitatively, by histochemical staining, and quantitatively, by biochemical assays of the gene product. The magnetically levitated plants showed evidence of reporter gene activation, however, the control plants placed in a static magnetic field of 19 Tesla showed similar patterns of transgene expression [21].

These observations lead to the design of additional experiments using transgenic plants as biomonitors of the effects of high magnetic fields in metabolically active tissues. The evaluation of global changes in the Arabidopsis genome in response to exposure to high magnetic fields was facilitated with Affymetrix Gene Chip microarrays. These arrays allow for the survey of over 8000 genes at a time and were used for genome-wide characterization of the effects of exposing Arabidopsis plants to a field of 21 Tesla. The resulting differential patterns of gene expression from the array data were then used to guide quantitative analyses of gene expression with quantitative real-time polymerase-chain-reaction (qRT-PCR), which is an effective means of characterizing an abiotic stress response [22]. The microarray data indicate that, of the 8000 genes surveyed, there were 114 genes that were differentially expressed to a degree greater than 2.5 fold over the control. These results were quantitatively corroborated by qRT-PCR examination of 4 of the 114 genes.

The data suggest that magnetic fields in excess of 15 Tesla have far-reaching effect on the genome. The wide-spread induction of stress-related genes and transcription factors, and a depression of genes associated with cell wall metabolism, are prominent examples. The roles of magnetic field orientation of macromolecules and magnetophoretic effects are discussed as possible factors that contribute to the mounting of this response.

## Materials and methods

### Magnetic fields and local environment control

Four experimental runs were made at the National High Magnetic Field Laboratory (NHMFL) in Tallahassee using the magnets housed in Cell 5 (Runs 1, 4 and 5) and in Cell 6 (Run 2). The general physical dimensions of these magnets and typical field profiles are available online [23]. The temperature of incoming/outgoing cooling water for the magnets was monitored and was regulated to provide an average temperature of 13 (± 3)°C. At a constant magnetic field, typically five plants, each individually grown in plastic tubes, were held in a 50 ml plastic beaker that was attached to a copper tubing coil assembly. After exposure for either 2.5 or 6.5 hours, two plants were stained and the other three plants were used for the quantitative, biochemical assays. Perforations in the bottom of the beaker allowed for a significant flux of air within the bore of the magnet at all field strengths. Thermally regulated water was circulated in the copper tubing, and the temperature near the bottom of the plastic beaker and near the leaves was monitored with Cu-CuNi thermocouples whose magneto-response had been calibrated. The thermocouples were monitored every 15 minutes during the course of a run, and the plants were maintained near 15 (± 3)°C after cooling from the ambient temperature, nominally 20°C. The leaves of the plants were spritzed with water before being inserted into the bore and the ambient humidity was sufficient to allow water to condense on the copper tubing. The plants were inspected and photographed after being removed from the magnet, and in no case was there any evidence of wilting effects caused by the flux of air through the bore of the magnet. The plants were inserted so that the center field was at the shoot/root boundary. Given the finite size of the plants and their location in the magnet, the largest field gradients that the plants experienced for the NHMFL experiments were approximately (0.5 B_0_) cm^-1^, where B_0 _is the magnetic field (in Tesla) at the center of the magnet. The ambient light down the bore of the magnet was sufficient to allow the plants to be seen without any difficulty and no additional lighting sources were used. A similar arrangement was used for an experiment (Run 3) performed with the superconducting solenoid located at the University of Florida. However, since the large bore (88 mm diameter) was accessible at room temperature, the cooling water assembly was not required, and the specimens always experienced ambient temperature. For this magnet, the largest field gradients that the plants experienced were approximately (10^-4 ^B_0_) cm^-1^. Experimental Runs 1 – 4 focused on the *Adh*/GUS studies, and Run 5 was performed to allow the microarray and qRT-PCR analyses.

### Plant treatments

A previously developed line of transgenic Arabidopsis (*Arabidopsis thaliana*) plants containing the *alcohol dehydrogenase *(*Adh*) gene promoter linked to the β-glucuonidase (GUS) gene reporter were used throughout. The histochemical analyses were conducted with 21 day-old plants grown on the slanted surface of 9 mm diameter tubes containing a nutrient agar medium. Plants intended for gene expression analyses were grown vertically on nutrient agar plates for 9 days before introduction to the magnetic field [19,22]. After exposure to the magnetic field, plants to be subjected to subsequent gene expression or biochemical analyses were harvested to liquid nitrogen, and plants intended for evaluations of tissue-specific transgene expression were fixed in histochemical stain. Control plants were treated similarly; the 21 day-old control plants for the histochemical and biochemical analyses were simply maintained on the lab bench for the duration of the magnetic field exposure. The 9 day-old plate plants being evaluated for gene expression changes were maintained in the bore of the magnet (with zero applied field) for an equivalent duration of time.

### Histochemical staining and biochemical assay of Adh/GUS expression

The histochemical stain is composed of the GUS substrate X-Gluc (2 mM 5-bromo-4-chloro-3-indolylglucuronide), 1% [w/v] dimethylformamide, 0.1 mM K_3 _[Fe(CN_6_)], 0.1 mM K_4 _[Fe(CN_6_)]·3H_2_O, 1 mM EDTA and 50 mM NaPO_4_, pH 7.0. Plants were harvested to stain immediately following treatment and incubated at room temperature 48 hours. The reaction was stopped and chlorophyll cleared from plants with several washes of 70% ethanol. Plants were then photographed to record tissue-specific deposition of the *Adh*/GUS transgene product. The samples frozen for biochemical analysis were homogenized in extraction buffer (50 mM NaPO_4_, pH 7.0, 10 mM EDTA, 0.1% sarkosyl, 0.1% [v/v] Triton X-100, and 10 mM β-mercaptoethanol) and diluted for incubation with a fluorometric substrate. The GUS activity in the lysate was measured quantitatively on a fluorometer (Shimadzu, Kyoto) as nanomoles of substrate (4 methylumbelliferyl β-D-glucuronide [4-MUG]) reacted with the GUS enzyme per microgram total protein per minute [24].

### Microarray sample preparation

Five 9 day-old plants (grown as indicated above) were selected at random from a pool of about 70 plants for each treatment. The selected plants were placed in the bore of the magnet, and a field of 21 Tesla was applied for 2.5 hours. The controls were conducted in the bore of the magnet in the absence of an applied field. The plants in the bore were controlled for temperature by circulating chilled water through copper coils within the plant container. The actual temperature was monitored via thermocouples and recorded manually (16 – 18°C). Plants were harvested to liquid nitrogen immediately upon removal from the bore and stored at -80°C until RNA isolation procedure. Three of the five plants exposed to 21 Tesla for 6.5 hours (and the comparable control) were combined, and total RNA was isolated from the pooled samples for microarray analyses. The RNA was extracted using RNAeasy™ kits from Qiagen [22]. Purified RNAs were labeled and prepared for hybridization according to the protocols outlined in the GeneChip^® ^Expression Analysis Technical Manual (Revision 1, 2001, Affymetrix, Santa Clara, Ca).

### Quantitative qRT-PCR

The two remaining plants from the five exposed to 21 Tesla for 6.5 hours were separated into root and shoot fractions and the tissue types pooled before RNA extraction (as previously described). In addition, five plants exposed to 14 Tesla for 2.5 hours and 21 Tesla for 6.5 hours were similarly treated, as were the remaining controls. Genes to be targeted for quantification were identified from the microarray results. The quantification was conducted with Taqman^® ^Real Time Reverse Transcriptase – Polymerase Chain Reaction (qRT-PCR) from Applied Biosystems (ABI) [25] and evaluated with the ABI Prism^® ^7700 Sequence Detection Systems. Forward and reverse primers flanking a ca. 100 bp region of the gene of interest were obtained from ABI along with the fluorescently labeled probe sequence that hybridizes between the primer pairs.

## Results

### Histochemical and biochemical indications

**Figure 1:**
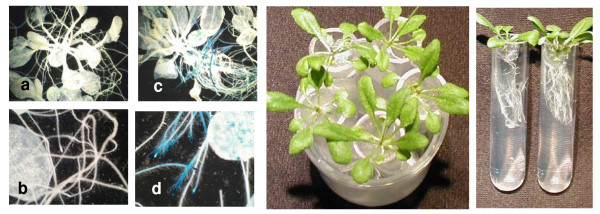
Qualitative examples of GUS expression. Histochemical staining of the controls indicates that *Adh*/GUS was not expressed in these plants (a – b). An increase in magnetic field strength induces expression of the *Adh*/GUS transgene (e.g. 20 Tesla for 2.5 hours, c – d). The increased magnification of the plants shown in b and d (second row) provide closer inspection of GUS localization in the roots and leaves of these samples. The middle panel (e) provides a top-view of the five 21 day-old plants just prior to insertion into the bore of the magnet. The right hand panel (f) shows the plants from the side.

**Figure 2:**
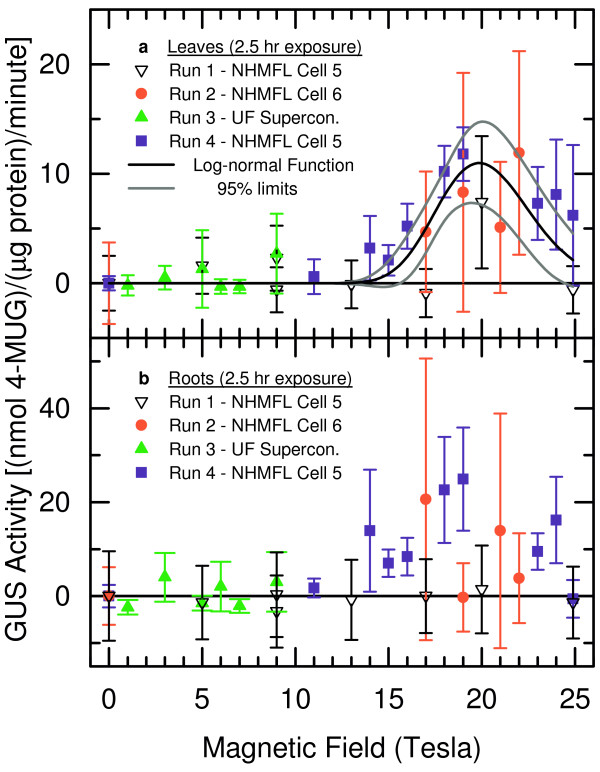
Quantitative GUS activity as a function of applied magnetic field. The amount of GUS activity, as measured by biochemical/fluorometric procedures, is plotted as a function of applied magnetic field. Each data point represents the average of three plants and the uncertainty limits are given by the standard deviation. Three data sets were collected at the NHMFL and one was obtained in a UF superconducting solenoid capable of reaching 9 Tesla. Nonparametric statistical methods have been used to test the hypothesis about an association between the GUS activity and the magnetic field. For the leaves (a), the Spearman correlation coefficient of 0.58 (*P *= 0.001) indicates that there exists a significant association between these two variables. For the roots (b), the Spearman correlation coefficient of 0.40 (*P *= 0.033) suggests a somewhat weaker association. A statistical model was formulated, hypothesizing a log-normal relationship for the effect of the magnetic field on the level of GUS activity (see text). The data for the leaves (a) were analyzed using nonlinear weighted least squares, and the result is plotted as the black line, with the 95% confidence limits given by the grey lines.

In a series of experiments, 21 day-old plants were exposed for durations of 2.5 hours per run in the 50 mm diameter bore resistive magnets at the National High Magnetic Field Laboratory (NHMFL) in Tallahassee, and in the 88 mm diameter bore superconducting solenoid located at the University of Florida. As described in the Materials and Methods section, the local environmental variables of temperature, light intensity, atmosphere, and humidity were controlled, and did not impact transgene expression. Figure 1 provides qualitative examples of the stress response mounted by the plants exposed to high magnetic fields. The histochemical staining shown in Figure 1 illustrates that GUS is being expressed in both leaf and root tissues in these plants. The baseline corrected, quantitative results are provided in Figure 2. Each data point in Figure 2 represents the average of the results from three plants, and the standard deviation is given by the uncertainty limits. Nonparametric statistical methods have been used to test the hypothesis of an association between the GUS activity and the magnetic field. For the leaves (Figure 2a), the Spearman correlation coefficient of 0.58 (*P *= 0.001) indicates that there exists a significant association between these two variables [26,27]. For the roots (Figure 2b), the Spearman correlation coefficient of 0.40 (*P *= 0.033) suggests a somewhat weaker association [26,27].

In a second step, a statistical model was formulated, hypothesizing a log-normal relationship (*y *= *A *exp [-ln^2^(*x*/*x*_*c*_)/2*w*^2^]) for the effect of the magnetic field (*x*) on the level of GUS activity (*y*). A log-normal functional form might be anticipated as the presence of the magnetic field, above a threshold value, initiates a stress response, while subsequently stronger magnetic fields further perturb all gene activation, causing the GUS activity to decline. The data for the leaves (Figure 2a) were analyzed using nonlinear weighted least squares [28,29], with the weights computed as the reciprocals of the variance estimates, yielding parameter estimates (± standard errors) of *A *= 11 ± 2, *x*_*c *_= 19.9 ± 0.4, and *w *= 0.12 ± 0.02. This result is plotted as the black line in Figure 2a, with the 95% confidence limits represented by the grey lines. Standard techniques (i.e. residual analysis, evaluation of correlation matrix, *R*^2 ^measure) were used for checking the goodness of fit of the model. Finally, it is important to note that neither simple-linear nor quadratic dependences are represented in the data. These two functional forms might be anticipated if the stress response was linked to some other magnetic field factor, e.g. a small amount of sinusoidal field ripple, rather than just the strength of the static field.

### Gene expression analyses

**Figure 3:**
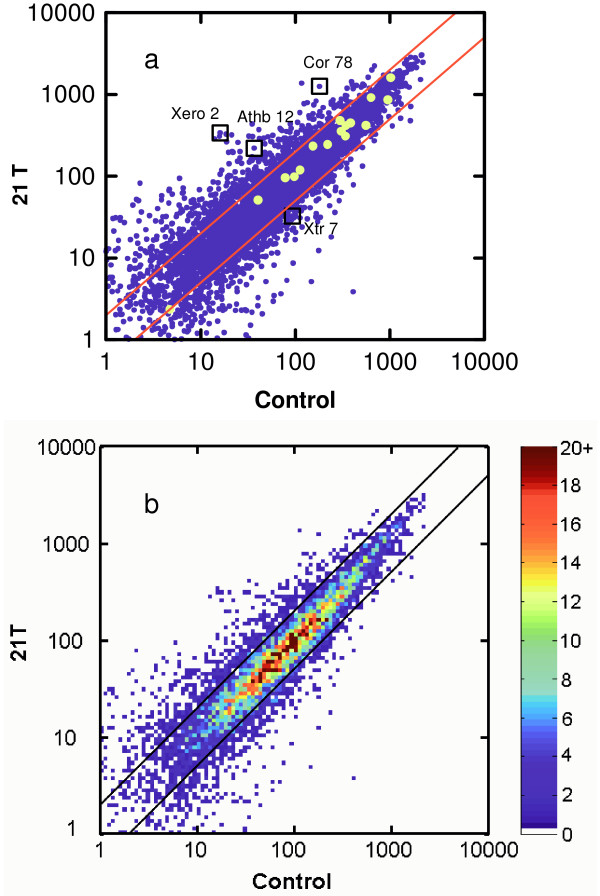
Scatterplots of the Affymetrix ATH1 Arabidopsis Array for magnetic field data. The 9 day-old plants were exposed to a field of 21 Tesla for 6.5 hours and compared to control plants that had been maintained in the bore of the magnet for the same amount of time without an applied field. In (a), each data point represents the level of differential expression for each of the 8000 genes represented on the microarray, and the axes are logarithmic. Genes of interest that were chosen for further quantification are indicated with boxes, see text. The large yellow spots represent "housekeeping" genes that are typically unaffected by abiotic stress. The two parallel lines represent the 2-fold increase or decrease limits. The data in (a) replotted in (b) using a topographical routine [30], where the color gradient designates the number of genes appearing in a given localized region of the graph.

Three week-old plants were exposed to field strengths of 14 Tesla for 2.5 hours and of 21 Tesla for durations of 2.5 and 6.5 hours. The control experiments were conducted in the bore of the magnet in the absence of an applied field. Figure 3 shows scatterplots comparing the gene expression patterns of plants exposed to 21 Tesla for 6.5 hours and control plants maintained in ambient field strength within the bore of the magnet for the equivalent amount of time. The levels of differential expression for the 8000 genes represented on the microarray are indicated by dots in Figure 3a, and regions of high density become saturated with data points. Figure 3b, a topographical (or "relief") version of Figure 3a, provides a sense of the density of the data points [30] and indicates that most of the 8000 representative genes show less than a 2-fold difference in expression between treatment and control. The yellow spots in Figure 3a represent genes that are typically unaffected by abiotic stress (i.e. "housekeeping" genes), and as expected, these housekeeping genes show little evidence of differential expression. There are, however, 114 genes that were differentially expressed to a degree greater than 2.5 fold over the control. In this group, many genes associated with a variety of stress responses were induced (heat, cold, drought, touch), as were genes encoding proteins that are involved with ion transport functions (chloride, sulfate, ammonium). The down regulated set included a number of genes involved in cell wall biosynthesis (e.g. Xtr7). A final large category is populated by genes that encode transcription factors (e.g. Athb12). A listing of all 114 genes is given in the supplementary material [See Additional file 1].

**Figure 4:**
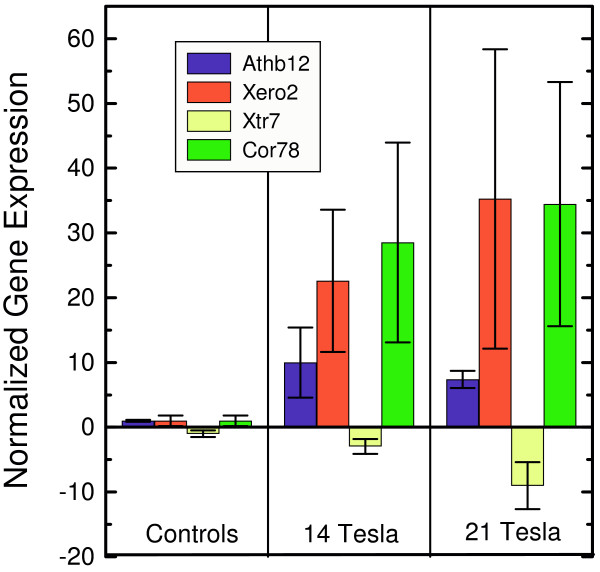
Quantitative qRT-PCR analyses of selected genes and treatments. Four genes were chosen for additional quantification: Athb12, Xero2, Xtr7, and Cor78. The normalized gene expression levels represent fold-induction or fold-repression relative to the controls. The exposure to the magnetic fields was for 2.5 hrs.

Quantitative qRT-PCR was used to corroborate the expression patterns of a select number of genes indicated by the arrays, and these genes are designated by the boxes in the scatterplot of Figure 3. Figure 4 shows the results the results of utilizing quantitative RT-PCR (ABI) to determine the differential expression levels of expression for four genes: Athb12 (AF001949, a homeobox transcription factor), Xero2 (U19536, a dehydrin), Xtr7 (U43489, xyloglucan endotransglycoslase) and Cor78 (L22567, cold regulated 78). Values within each treatment are depicted as fold induction or repression relative to the control. The controls represent plants held within the bore of the magnet without the application of an exogenous magnetic field. All conditions for the controls and the subsequent runs were identical, with the exception of field strength, and the exposure time was 2.5 hours. The genes Athb12, Xero2 and Cor78 display an increase in the level of expression when in a strong magnetic field, whereas expression of Xtr7 decreases with increasing field. The patterns of gene expression determined with quantitative qRT-PCR, Figure 4, reflect the trends in expression indicated by the array data, Figure 3.

## Discussion

The results indicate that high magnetic fields have far-reaching effects on the genome. The biological impact of high magnetic fields on Arabidopsis, as quantified by microarray and qRT-PCR analyses, is stronger than was reported for microarry data obtained for TCA cycle-related genes of budding yeast (*Saccharomyces cerevisiae*) exposed to fields up to 14 Tesla [31]. Although a detailed understanding of the results will require additional studies, perhaps involving isolated *in vitro *processes [32], hints of the underlying mechanism generating the effects may be gleaned from the strength of the response as a function of applied magnetic field. For example, the data in Figures 2 and 4 suggest that while a minimum threshold field can initiate a stress response that is manifested as either an induction or a repression of select genes, higher fields may compromise some aspects of the transcriptional machinery, and effectively arrest the process. This field dependence may suggest that magnetic orientation or magnetophoresis plays a role in the seemingly dual nature of the response.

An order of magnitude comparison between the strength of magnetic orientation and magnetophoresis can be made for the experimental conditions of our experiments. It is important to stress that although these two effects are present in our experiments, the current results do not identify them as the source of the effects on gene expression. However, these types of effects have been detected in experimental configurations evaluating the magnetic orientation or magnetophoresis on molecules, so they are relevant for the present discussion. Specifically, the biomacromolecules involved in signal transduction and gene regulation may experience forces and/or torques that are induced by the presence of the magnetic field. For example, one such torque arises from the anisotropy of the diamagnetic susceptibility [33,34] of the molecule and attempts to magnetically orient the macromolecule. This effect has been known for some time, but it has only recently been exploited in NMR structural determinations of large molecules as the measurements evolved from 14 Tesla to 17.5 Tesla [35,36]. In addition, the macromolecules may experience magnetophoresis due to forces generated by inhomogeneities in the applied magnetic field.

More specifically, in a magnetic field B→
 MathType@MTEF@5@5@+=feaafiart1ev1aaatCvAUfKttLearuWrP9MDH5MBPbIqV92AaeXatLxBI9gBaebbnrfifHhDYfgasaacH8akY=wiFfYdH8Gipec8Eeeu0xXdbba9frFj0=OqFfea0dXdd9vqai=hGuQ8kuc9pgc9s8qqaq=dirpe0xb9q8qiLsFr0=vr0=vr0dc8meaabaqaciaacaGaaeqabaqabeGadaaakeaacuWGcbGqgaWcaaaa@2DCB@(r→
 MathType@MTEF@5@5@+=feaafiart1ev1aaatCvAUfKttLearuWrP9MDH5MBPbIqV92AaeXatLxBI9gBaebbnrfifHhDYfgasaacH8akY=wiFfYdH8Gipec8Eeeu0xXdbba9frFj0=OqFfea0dXdd9vqai=hGuQ8kuc9pgc9s8qqaq=dirpe0xb9q8qiLsFr0=vr0=vr0dc8meaabaqaciaacaGaaeqabaqabeGadaaakeaacuWGYbGCgaWcaaaa@2E2B@), where r→
 MathType@MTEF@5@5@+=feaafiart1ev1aaatCvAUfKttLearuWrP9MDH5MBPbIqV92AaeXatLxBI9gBaebbnrfifHhDYfgasaacH8akY=wiFfYdH8Gipec8Eeeu0xXdbba9frFj0=OqFfea0dXdd9vqai=hGuQ8kuc9pgc9s8qqaq=dirpe0xb9q8qiLsFr0=vr0=vr0dc8meaabaqaciaacaGaaeqabaqabeGadaaakeaacuWGYbGCgaWcaaaa@2E2B@ is the vector identifying the spatial coordinates, the magnetic energy of an object possessing a magnetic susceptibility tensor χ↔
 MathType@MTEF@5@5@+=feaafiart1ev1aaatCvAUfKttLearuWrP9MDH5MBPbIqV92AaeXatLxBI9gBaebbnrfifHhDYfgasaacH8akY=wiFfYdH8Gipec8Eeeu0xXdbba9frFj0=OqFfea0dXdd9vqai=hGuQ8kuc9pgc9s8qqaq=dirpe0xb9q8qiLsFr0=vr0=vr0dc8meaabaqaciaacaGaaeqabaqabeGadaaakeaaiiaacuWFhpWygaqdaaaa@2E7C@ may be written as [36,37]

E=−12μB→(r→)⋅χ↔⋅B→(r→),     (1)
 MathType@MTEF@5@5@+=feaafiart1ev1aaatCvAUfKttLearuWrP9MDH5MBPbIqV92AaeXatLxBI9gBaebbnrfifHhDYfgasaacH8akY=wiFfYdH8Gipec8Eeeu0xXdbba9frFj0=OqFfea0dXdd9vqai=hGuQ8kuc9pgc9s8qqaq=dirpe0xb9q8qiLsFr0=vr0=vr0dc8meaabaqaciaacaGaaeqabaqabeGadaaakeaacqWGfbqrcqGH9aqpcqGHsisldaWcaaqaaiabigdaXaqaaiabikdaYiabeY7aTbaacuWGcbGqgaWcaiabcIcaOiqbdkhaYzaalaGaeiykaKIaeyyXICTafq4XdmMba0aacqGHflY1cuWGcbGqgaWcaiabcIcaOiqbdkhaYzaalaGaeiykaKIaeiilaWIaaCzcaiaaxMaadaqadaqaaiabigdaXaGaayjkaiaawMcaaaaa@46F6@

where μ is the permittivity of the material and a reasonable approximation is that μ = μ_0_, the permittivity of free space. Variations of this energy arise from anisotropies of the susceptibility and of the magnetic field, such that the dominant effects are given by

δE=−12μ0{B→(r→)⋅δχ↔⋅B→(r→)+2B→(r→)⋅Δχ↔⋅δB→(r→)}.     (2)
 MathType@MTEF@5@5@+=feaafiart1ev1aaatCvAUfKttLearuWrP9MDH5MBPbIqV92AaeXatLxBI9gBaebbnrfifHhDYfgasaacH8akY=wiFfYdH8Gipec8Eeeu0xXdbba9frFj0=OqFfea0dXdd9vqai=hGuQ8kuc9pgc9s8qqaq=dirpe0xb9q8qiLsFr0=vr0=vr0dc8meaabaqaciaacaGaaeqabaqabeGadaaakeaacqaH0oazcqWGfbqrcqGH9aqpcqGHsisldaWcaaqaaiabigdaXaqaaiabikdaYiabeY7aTnaaBaaaleaacqaIWaamaeqaaaaakmaacmqabaGafmOqaiKbaSaacqGGOaakcuWGYbGCgaWcaiabcMcaPiabgwSixlabes7aKjqbeE8aJzaanaGaeyyXICTafmOqaiKbaSaacqGGOaakcuWGYbGCgaWcaiabcMcaPiabgUcaRiabikdaYiqbdkeaczaalaGaeiikaGIafmOCaiNbaSaacqGGPaqkcqGHflY1cqqHuoarcuaHhpWygaqdaiabgwSixlabes7aKjqbdkeaczaalaGaeiikaGIafmOCaiNbaSaacqGGPaqkaiaawUhacaGL9baacqGGUaGlcaWLjaGaaCzcamaabmaabaGaeGOmaidacaGLOaGaayzkaaaaaa@617A@

The first term on the right hand side of Equation 2 is the energy due to anisotropy of the magnetic susceptibility and causes macromolecules to orient to minimize this energy. The second term on the right hand side of Equation 2 is the energy due to inhomogeneities in the magnetic field and differences of the susceptibilities of the molecules and their surroundings. In order to provide an order of magnitude comparison of the sizes of the two effects, the ratio, R, of these two terms may be written as

R=2B→⋅Δχ↔⋅δB→B→⋅δχ↔⋅B→≈2ΔχδBδχB.     (3)
 MathType@MTEF@5@5@+=feaafiart1ev1aaatCvAUfKttLearuWrP9MDH5MBPbIqV92AaeXatLxBI9gBaebbnrfifHhDYfgasaacH8akY=wiFfYdH8Gipec8Eeeu0xXdbba9frFj0=OqFfea0dXdd9vqai=hGuQ8kuc9pgc9s8qqaq=dirpe0xb9q8qiLsFr0=vr0=vr0dc8meaabaqaciaacaGaaeqabaqabeGadaaakeaacqqGsbGucqGH9aqpdaWcaaqaaiabikdaYiqbdkeaczaalaGaeyyXICTaeuiLdqKafq4XdmMba0aacqGHflY1cqaH0oazcuWGcbGqgaWcaaqaaiqbdkeaczaalaGaeyyXICTaeqiTdqMafq4XdmMba0aacqGHflY1cuWGcbGqgaWcaaaacqGHijYUdaWcaaqaaiabikdaYiabfs5aejabeE8aJjabes7aKjabdkeacbqaaiabes7aKjabeE8aJjabdkeacbaacqGGUaGlcaWLjaGaaCzcamaabmaabaGaeG4mamdacaGLOaGaayzkaaaaaa@5759@

The inhomogenetiy of the magnetic field is largest at the top of the leaves and the bottom of the roots where (δ*B*/*B*) ≈ 5 × 10^-3^. On the other hand, a reasonable bound for the anisotropy of the susceptibility for biomacromolecules is 10^-1 ^< (δχ/Δχ) < 1 [1,15,36-38]. Consequently, 10^-2 ^< R < 10^-1^, and the effect of magnetic orientation dominates the magnetophoretic effects in our experiments. Although the sum of the variations of the magnetic energy is less than 100 ppm of the ambient thermal energy (= *k*_B_*T*, where *k*_B _is the Boltzmann constant and *T *is the temperature ≈ 290 K), the magnetic orientation is readily observable [1,36-38], and magnetophoretic effects, arising from *B*δ*B *ranges similar to the ones present in our work, have been reported [39].

## Conclusion

To summarize, although each of these magnetic-field-induced effects is quite small compared to the randomizing ambient thermal energy of the plant, we conjecture that they are sufficient to perturb the delicate conformational dynamics involved in aspects of gene regulation, thereby resulting in the differential expression of a variety of genes in the plant. Compared to each other, the magnetic orientation effects are estimated to be 10 to 100 times larger than the magnetophoretic forces for our experiments, thus it is likely that magnetophoresis plays a minor role in the induction or repression of gene expression. Our results indicate that nominally 15 Tesla is the threshold of field strength required to initiate the stress response. It is interesting to note that reports of magnetic orientation effects during NMR studies of macromolecules intensified when the fields were increased from 14 Tesla to 17.5 Tesla [35,36]. These observations are consonant with the idea that macromolecular orientation plays a role in stress-gene activation in field strengths greater than 15 Tesla. On a macroscopic scale, static magnetic fields of 10 Tesla are sufficient to align the cleavage planes in developing frog eggs [7-9]. However, on the basis of our results, we cannot determine if the observed effects are related to assemblies of molecules or simple conformational flexing/bending of single molecules. There are other processes (e.g. chemical reactions [40]) that might be perturbed by the presence of the magnetic field, and these perturbations may impact local chemistry of any of several signaling pathways that are connected to the differential expression of any number of genes.

The induction of the *Adh*/GUS transgene was the first indication that gene expression processes may be sensitive to high magnetic fields. The GUS gene induction is a highly sensitive histochemical reaction that can indicate even subtle tissue-specific changes in gene expression. Subsequent experiments surveyed genome-wide changes in gene expression in response to exposing Arabidopsis plants to 21 Tesla. Of the 8000 genes surveyed, there were 114 genes that were differentially expressed to a degree greater than 2.5 fold over the control.

In conclusion, exposure to magnetic fields above nominally 15 Tesla is accompanied by differential gene expression responses in Arabidopsis plants. These data provide evidence for the perturbation of metabolic processes in the presence of strong magnetic fields and may be useful for guiding future research designed to calibrate safe exposure standards for living organisms [5].

## Abbreviations

Adh: alcohol dehydrogenase

Athb12: AF001949, a homeobox transcription factor

Cor78: L22567, cold regulated 78

EDTA: ethylenediaminetetraacetic acid

GUS: β-glucuronidase

NHMFL: National High Magnetic Field Laboratory

NMR: nuclear magnetic resonance

MRI: magnetic resonance imaging.

TAGES: Transgenic Arabidopsis Gene Expression System

TCA: tricarboxylic acid

qRT-PCR: quantitative real-time polymerase-chain-reaction

Xero2: U19536, a dehydrin

Xtr7: U43489, xyloglucan endotransglycoslase

## Competing interests

The author(s) declare that they have no competing interests.

## Authors' contributions

ALP and RJF designed and executed the work directly related to the plants and the associated analyses. MWM designed the magnetic studies and generated the analysis related to the comparison of the competing magnetic interactions. ALP and MWM drafted the manuscript, and all authors read and approved the final version.

## Supplementary Material

Additional file 1 Supplementary Material. Excel Worksheet, and two plots clustered by function, of the 114 genes that were differentially expressed, between treatment in 21 Tesla and zero-field bore controls, to a degree greater than 2.5 fold. Plants were held at 21 Tesla or at zero-field, in the bore of the magnet, for 6.5 hours. (Paul-Ferl-Meisel-Supplementary-Material.xls) Click here for file
